# EZH2 expression is associated with inferior overall survival in mantle cell lymphoma

**DOI:** 10.1038/s41379-021-00885-9

**Published:** 2021-08-10

**Authors:** Diana Martinez-Baquero, Ali Sakhdari, Huan Mo, Do Hwan Kim, Rashmi Kanagal-Shamanna, Shaoying Li, Ken H. Young, Dennis P. O’Malley, Ahmet Dogan, Preetesh Jain, Michael L. Wang, Timothy J. McDonnell, Roberto N. Miranda, Francisco Vega, L. Jeffrey Medeiros, Chi Young Ok

**Affiliations:** 1 Department of Hematopathology, The University of Texas M.D. Anderson Cancer Center, Houston, TX, USA.; 2 Department of Pathology, Faculty of Medicine, Universidad Nacional de Colombia, Bogotá, Colombia.; 3 Department of Laboratory Medicine and Pathobiology, University Health Network, The University of Toronto, Toronto, ON, Canada.; 4 Division of Hematopathology and Department of Pathology, Duke University School of Medicine, Durham, NC, USA.; 5 NeoGenomics, Aliso Viejo, CA, USA.; 6 Memorial Sloan Kettering Cancer Center, New York, NY, USA.; 7 Department of Lymphoma and Myeloma, The University of Texas M.D. Anderson Cancer Center, Houston, TX, USA.; 8 These authors contributed equally: Diana Martinez-Baquero, Ali Sakhdari.

## Abstract

Enhancer of zeste homolog 2 (EZH2) is a catalytic component of the polycomb repressive complex 2 (PRC2) which reduces gene expression via trimethylation of a lysine residue of histone 3 (H3K27me3). Expression of EZH2 has not been assessed systematically in mantle cell lymphoma (MCL). Expression of EZH2 was assessed by immunohistochemistry in 166 patients with MCL. We also assessed other PRC2 components and H3K27me3. Fifty-seven (38%) of MCL patients were positive for EZH2 using 40% cutoff. EZH2 expression was associated with aggressive histologic variants (65% vs. 29%, *p* < 0.001), high Ki-67 proliferation rate (median, 72% vs. 19%, *p* < 0.001), and p53 overexpression (43% vs. 2%, *p* < 0.001). EZH2 expression did not correlate with expression of other PRC2 components (EED and SUZ12), H3K27me3, MHC-I, and MHC-II. Patients with EZH2 expression (EZH2+) had a poorer overall survival (OS) compared with patients without EZH2 expression (EZH2−) (median OS: 3.9 years versus 9.4 years, respectively, *p* < 0.001). EZH2 expression also predicted a poorer prognosis in MCL patients with classic histology (median OS, 4.6 years for EZH2+ and 9.6 years for EZH2-negative, respectively, *p* < 0.001) as well as aggressive histology (median OS, 3.7 years for EZH2+ and 7.9 years for EZH2− negative, respectively, *p* = 0.046). However, EZH2 expression did not independently correlate with overall survival in a multivariate analysis. Gene expression analysis and pathway enrichment analysis demonstrated a significant enrichment in cell cycle and mitotic transition pathways in MCL with EZH2 expression. EZH2 expression detected by immunohistochemistry is present in 38% of MCL cases and it is associated with high proliferation rate, p53 overexpression, aggressive histologic variants, and poorer OS. Based on gene expression profiling data, EZH2 expression could potentiate cell cycle machinery in MCL. These data suggest that assessment of EZH2 expression could be useful to stratify MCL patients into low- and high-risk groups.

## INTRODUCTION

Mantle cell lymphoma (MCL) is an aggressive type of B-cell lymphoma characterized by t(11;14)(q13;q32) resulting in overexpression of cyclin D1^1^. Patients tend to be older men, with a median age at diagnosis of 68 years and a male-to-female ratio of 3:1. Proliferation rate, usually assessed by immunohistochemical assessment of Ki-67, is an important prognostic factor in MCL and is incorporated into the Mantle Cell Lymphoma-International Prognostic Index (MIPI) to add more discriminative power in risk stratification^2^. Risk-adapted therapies can be planned for individual MCL patients. This type of lymphoma was regarded as more aggressive than other types of small B-cell lymphomas with a median survival of 3–5 years. However, recent therapeutic regimens, including rituximab, cyclophosphamide, vincristine, doxorubicin and dexamethasone (R-hyperCVAD) or the Nordic regimen with autologous stem cell transplant, have improved median survival to 8–10 years^3,4^.

Polycomb group proteins (PcG) are evolutionarily conserved regulators of gene silencing, important in stem cell pluripotency, cell development and X chromosome inactivation^5^. PcG proteins form multiprotein complexes designated as polycomb repressive complex 1 (PRC1) and PRC2. Key components of PRC2 include enhancer of zeste homolog 2 (EZH2), embryonic ectoderm development (EED), suppressor of zeste 12 (SUZ12), and RbAp46/48. EZH2 provides PRC2 with its catalytic activity but requires the remaining subunits to be fully functional. The PRC2 induces trimethylation of histone H3 at lysine 27 (H3K27me3), leading to gene repression^6^. On the other hand, loss of PRC2 enzymatic activity results in the acetylation of H3K27, promoting gene activation^7^. Dysregulation of *EZH2* was first linked to cancer by performing gene expression profiling in prostate cancer, which showed EZH2 is involved in prostate cancer progression, and its expression distinguishes indolent forms of prostate cancer from those forms at risk of lethal progression^8^. Similarly, EZH2 expression was associated with tumor aggressiveness and invasive potential in breast cancer^9^. In lymphomas, *EZH2* gain-of-function mutation is reported in about a quarter of follicular lymphoma (FL) cases, and in about 20% of diffuse large B-cell lymphoma of germinal center B-cell-like type^10,11^. Recently, trials treating FL patients with an EZH2 inhibitor yielded promising results, supporting the hypothesis that EZH2 is targetable when dysregulated^12^. However, *EZH2* mutation has not been reported in studies of MCL^13–22^.

Our group has observed that EZH2 expression as determined by immunohistochemistry correlates with Ki-67 proliferation rate in MCL^23^. However, our study was relatively small scale with 37 patients and was focused on cutaneous MCL. In literature, there are only a few studies available that assessed EZH2 expression in MCL^24–26^. Therefore, we conducted a systematic study of EZH2 expression in large cohort of MCL cases to assess its prevalence in MCL. We also correlated EZH2 expression with proliferation rate, expression of other PRC2 components and H3K27me3, some genomic markers background and clinical outcome.

## MATERIALS AND METHODS

### Patients

We searched the archives of the Department of Hematopathology at The University of Texas MD Anderson Cancer Center, Houston, Texas from July 1, 1997 through December 31, 2018 for cases of MCL obtained by excisional lymph node biopsy or resection of extranodal neoplasm for which formalin-fixed, paraffin-embedded tissue blocks were available. All patients fulfilled the diagnostic criteria for MCL based on the World Health Organization (WHO) classification system including *CCND1-IGH* translocation detected by fluorescence in situ hybridization or overexpression of cyclin D1 and all patients had MCL as a primary diagnosis. Cases of the leukemic, non-nodal variant of MCL were not included in this study. Clinical and laboratory data were collected by searching the electronic medical record. The MIPI was assessed using the formula suggested by Hoster et al.^27^. This study was conducted in accord with the Declaration of Helsinki and was approved by the institutional review board at The University of Texas MD Anderson Cancer Center, Houston, Texas^28^.

### Morphologic review

Histopathologic features were evaluated as classic versus aggressive (blastoid/pleomorphic) variants following the current WHO criteria. The architectural pattern was assessed as diffuse, nodular, or mantle zone pattern when a single pattern predominated (>75% involvement in tissue), similar to the approach of Hoster et al.^29^.

### Tissue microarray and immunohistochemistry

Hematoxylin-eosin stained slides from each MCL case were reviewed, and tumor-rich areas were selected. Tissue microarrays (TMAs) were constructed using a tissue microarrayer (Beecher Instrument, Silver Spring, MD). Two 1 mm cores were collected from each patient when possible (89%, 148 of 166 patients). When available tissue from the FFPE block is insufficient, only one 1 mm core was collected (11%, 18 of 166 patients). Immunohistochemical analyses were performed on 4 μm TMA sections using a streptavidin-biotin complex technique with antibodies reactive for the following antigens: CD5 and MHC-I (Thermo Fischer, Waltham, MA), CD10 (Leica Biosystems Inc., Lincolnshire, IL), CD20, Ki-67, and p53 (Dako/Agilent, Santa Clara, CA), cyclin D1 (Lab Vision, Freemont, CA), EZH2 (clone: D2C9), and H3K27me3 (Cell Signaling Technology, Danvers, MA), EED and SUZ12 (Abcam, Cambridge, United Kingdom) and SOX11 (clone: MRQ-58, Cell Marque, Rocklin, CA). MHC-II antibody (clone: LGII-612.4) was used courtesy of Dr. S. Ferrone (Massachusetts General Hospital, Boston, MA). Because of tissue exhaustion, staining was not always available for each marker. All stains were manually evaluated with 10% increment (from 0 to 100%) by AS and CYO without knowledge of other biomarkers (Ki-67, p53, morphologic variants) or survival. A conventional cutoff value for individual markers was decided upon based on previous reports in the literature. Receiver-operating characteristic (ROC) curves and X-tile analyses were used to assess an adequate cutoff for EZH2 expression. The cutoff score for these markers was as follows: 10% for CD5, CD10, and SOX11; 30% for Ki-67 (high vs. low), EED, and SUZ12; 40% for EZH2; and 50% for p53 overexpression^29,30^. Significant loss of MHC-I and MHC-II expression was determined when lymphoma cells showed only weak or no expression following the method of Ennishi et al.^31^.

### Gene expression profiling

Gene expression profiling was performed on 37 patients (17 and 20 patients for EZH2+ and EZH2(−) MCL, respectively) using RNA derived from formalin-fixed paraffin-embedded tissue and by using the nCounter PanCancer Pathway Panel (NanoString Technologies, Seattle, WA). The data quality control and normalization are performed on nSolver Analysis Software 4.0 (NanoString Technologies) with the default setting. Flagged specimens were removed from subsequence analysis. The normalized counts are log-transformed (base 10).

For each gene, the expression levels between EZH2+ and EZH2− negative MCLs were compared with a two-tailed Student’s *t* test for independent groups on KNIME 4.3.0. Statistical significance was defined by using Bonferroni method corrected cutoffs with the numbers of tests (*α* = 0.05/total genes tested = 6.38 × 10^−5^).

Pathway enrichment analyses were performed on reactome.org with default settings. Gene sets were downloaded from Kyoto Encyclopedia of Genes and Genomes (KEGG.org, accessed on 2/4/2021).

### Statistical analysis

Continuous variables were evaluated using the Mann–Whitney test to evaluate differences between datasets. Categorical variables were analyzed by Chi-square test. Overall survival (OS) was calculated from the day of diagnosis to the last follow-up. For patients who received hematopoietic stem cell transplant, survival was censored on the day of the procedure. Distributions of OS were estimated by Kaplan and Meier curves and survival differences were evaluated using the log-rank test. A *p* value (two-sided) < 0.05 was considered significant. Statistical analyses were performed using R, GraphPad Prism v8.0.0 (La Jolla, CA), and IBM SPSS Statistics Version 23 (Armonk, NY).

## RESULTS

### Study cohort

A total of 166 patients with MCL was identified, including 137 (83%) men and 29 (17%) women with a median age of 61 years (range, 39–88 years). Older (age ≥60 years) and younger patients were 91 (55%) and 75 (45%), respectively. Involved organs included lymph nodes (*n* = 125, 75%), spleen (*n* = 5, 3%), tonsils (*n* = 14, 8%), and other extranodal sites (*n* = 22, 13%). Thirteen (8%) and 146 (92%) patients had limited (I/II) or advanced (III/IV) stage, respectively. Using the MIPI score, 59 (45%), 42 (32%), and 29 (22%) patients were classified as low-, intermediate-, and high-risk, respectively. Ninety-nine (60%) patients had classic variant histology and 67 (40%) patients aggressive (blastoid or pleomorphic) histology. Nodular, diffuse, and mantle zone patterns were observed in 76 (46%), 88 (53%), and 2 (1%) patients, respectively. Patients were treated with hyperCVAD with or without rituximab (*n* = 106); cyclophosphamide, doxorubicin, vincristine, and prednisone (CHOP) with or without rituximab (*n* = 31); and bendamustine with rituximab (*n* = 5). Fourteen patients were observed, and treatment information was not available in 10 patients. Treatment response was available for 142 patients: 109 patients had a complete remission (CR), and 8, 5, and 11 patients had partial remission, stable disease, and progressive disease, respectively. In the 109 patients with CR, 68 patients (62.4%) eventually relapsed.

### EZH2 expression is associated with aggressive histology, high proliferation rate, and p53 overexpression

**Fig. 1:**
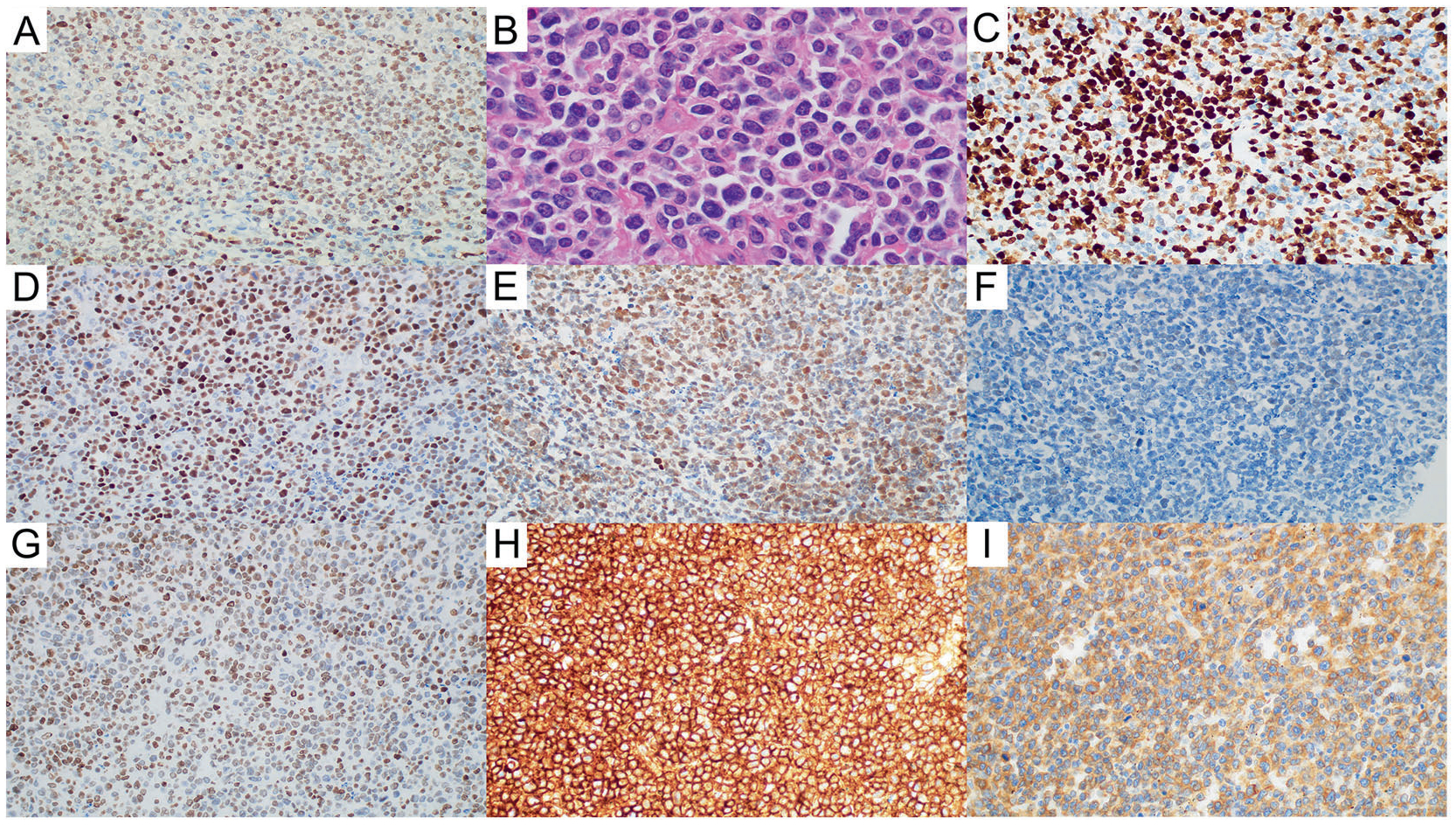
Mantle cell lymphoma (MCL) with EZH2 expression (EZH2+ MCL). **A** EZH2 expression (EZH2, ×40). **B** EZH2+ MCL is associated with MCL with aggressive morphology (Hematoxylin and eosin, ×100 with oil). **C** High Ki-67 proliferation rate in EZH2+ MCL (Ki-67, ×40). **D** p53 overexpression in EZH2+ MCL (p53, ×40). **E** EED expression in EZH2+ MCL (EED, ×40). **F** SUZ12 expression in EZH2+ MCL (SUZ12, ×40). **G** Expression of H3K27 trimethylation (H3K27me3) in EZH2+ MCL (H3K27me3, ×40). **H** MHC-I expression in EZH2+ MCL (MHC-I, ×40). **I** MHC-II expression in EZH2+ MCL (MHC-II, ×40).

**Fig. 2:**
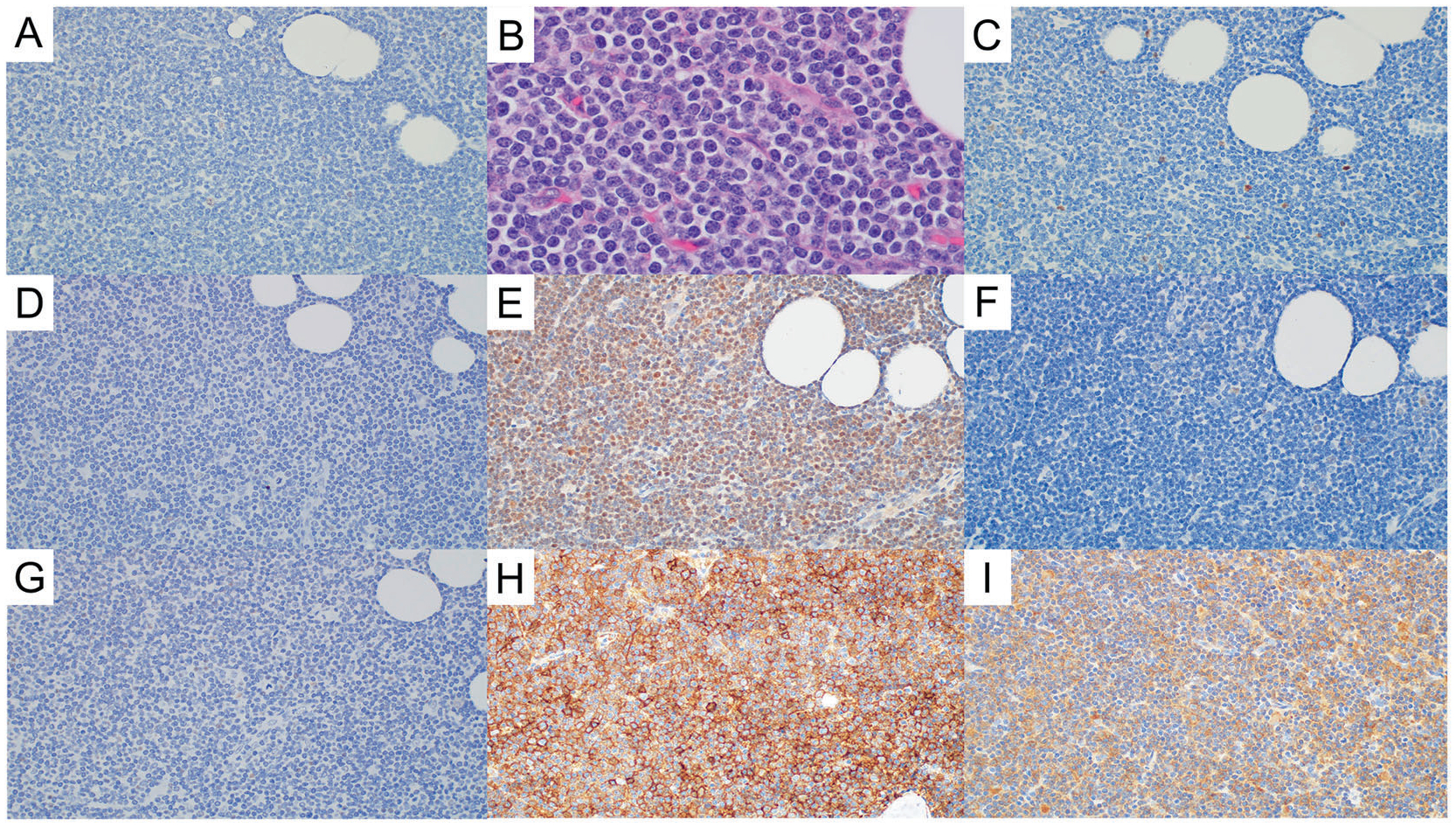
Mantle cell lymphoma without EZH2 expression (EZH2(−) MCL. **A** No EZH2 expression (EZH2, ×40). **B** EZH2(−) MCL is associated with MCL with classic morphology (Hematoxylin and eosin, ×100 with oil). **C** Low Ki-67 proliferation rate in EZH2(−) MCL (Ki-67, ×40). **D** No p53 overexpression in EZH2(–) MCL (p53, ×40). **E** EED expression in EZH2(−) MCL (EED, ×40). **F** SUZ12 expression in EZH2(−) MCL (SUZ12, ×40). **G** Expression of H3K27 trimethylation (H3K27me3) in EZH2(−) MCL (H3K27me3, ×40). **H** MHC-I expression in EZH2(−) MCL (MHC-I, ×40). **I** MHC-II expression in EZH2(−) MCL (MHC-II, ×40).

EZH2 expression was assessed in 150 patients (16 cases lost during TMA construction). Overall, EZH2 expression (≥40%) was found in 57 (38%) patients. Compared with EZH2-negative MCL, EZH2+ MCL was associated with aggressive histologic variants (65% vs. 29%, *p* < 0.001), a high Ki-67 proliferation rate (median, 72% vs. 19%, *p* < 0.001), and p53 overexpression (43% vs. 2%, *p* < 0.001) (Figs. 1, 2 and Table 1). Using the Pearson correlation test, EZH2 expression correlated with high Ki-67 proliferation rate (*r* = 0.732, *p* < 0.0001) and moderately correlated with p53 overexpression (*r* = 0.598, *p* < 0.001) (Supplementary Figs. 1, 2). Cases of EZH2+ MCL did not show distinctive features in terms of gender, age, involved sites, stage, MIPI score, growth pattern, or expression of SOX11, CD5, and CD10.

### EZH2 expression shows a weak correlation with other PRC2 complex molecules, but no correlation with H3K27me3 expression, or loss of MHC I/II

EZH2 expression was compared with other components (EED and SUZ12) of the PRC2 complex. Using the Pearson correlation test, EZH2 expression weakly correlated with SUZ12 (*r* = 0.372) and EED (*r* = 0.347) expression. EZH2 expression showed no correlation with H3K27me3 expression (*r* = 0.169). There was also no correlation between EZH2 expression and loss of MHC-I or MHC-II (*r* = −0.09 and *r* = 0.06, respectively) (Figs. 1, 2 and Supplementary Figs. 1, 2).

### EZH2 expression, but not EED and SUZ12, is associated with poor overall survival

**Fig. 3:**
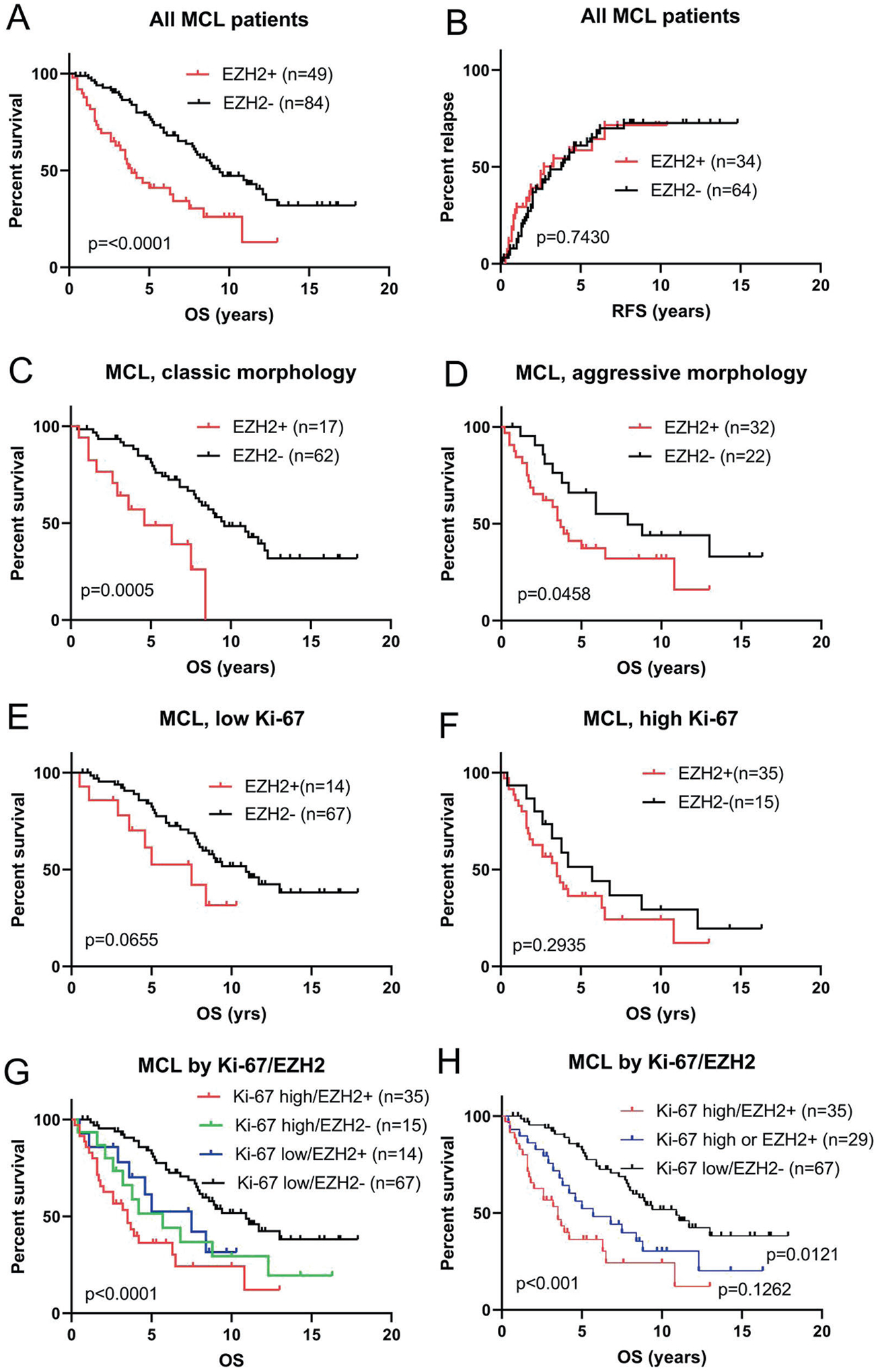
Kaplan–Meier curves in mantle cell lymphoma. **A** Overall survival (OS) with respect to EZH2 expression in all patients. **B** Relapse-free survival with respect to EZH2 expression in all patients. **C** Overall survival (OS) with respect to EZH2 expression in MCL patients with classic morphology. **D** Overall survival (OS) with respect to EZH2 expression in MCL patients with aggressive morphology. **E** Overall survival (OS) with respect to EZH2 expression in MCL patients with low (<30%) Ki-67 proliferation rate. **F** Overall survival (OS) with respect to EZH2 expression in MCL patients with high (≥30%) Ki-67 proliferation rate. **G** Overall survival (OS) with respect to EZH2 and Ki-67 proliferation rate in all MCL patients. **H** Overall survival (OS) with respect to EZH2 and Ki-67 proliferation rate in three groups.

A total of 148 patients had available survival data with a median follow-up interval of 5.3 years (range, 0.2–18.3 years). Patients with EZH2 expression (EZH2+) demonstrated inferior OS compared with patients without EZH2 expression (EZH2-negative) (median OS: 3.9 years and 9.4 years, respectively, *p* < 0.001) (Fig. 3A). However, patients with EZH2+ MCL did not show a significantly different relapse-free survival (RFS) from patients with EZH2− negative MCL (median RFS, 3.8 and 2.7 years, respectively, *p* = 0.7430) (Fig. 3B). Since our patient cohort include differently treated patients, we also performed subgroup analysis based on treatment regimen. In the group of patients treated with hyperCVAD ± rituximab (75% of our cohort), EZH2+ patients showed inferior OS then EZH2(−) patients (median OS: 5 years and 11.7 years, respectively, *p* = 0.0008). In the group treated with CHOP ± rituximab (22% of our cohort), a similar trend was observed (median overall survival: 3.5 years vs. 5.2 years, respectively, *p* = 0.0721) (Supplementary Fig. 3A, B).

Since EZH2 expression was associated with aggressive histology, we further analyzed this variable. In MCL with classic histology, patients with EZH2+ MCL had a poorer outcome compared with patients with EZH2-negative MCL (median, 4.6 and 9.6 years, respectively, *p* < 0.001) (Fig. 3C). In MCL with aggressive histologic variants, patients with EZH2+ MCL had a poorer outcome than patients with EZH2-negative MCL (median OS, 3.7 and 7.9 years, respectively, *p* = 0.0458) (Fig. 3D). Similarly, in patients with low Ki-67 MCL, EZH2+ MCL showed a trend toward a poorer prognosis (*p* = 0.0655) (Fig. 3E). In patients with high Ki-67 MCL, EZH2 expression did not correlated with OS (*p* = 0.2935) (Fig. 3F). We also assessed OS combining both Ki-67 and EZH2 (Fig. 3G). Patients with high Ki-67/EZH2+ MCL had the worst outcome and patients with high Ki-67/EZH2-negative MCL or low Ki-67/EZH2+ MCL mostly showed overlapping curves. Therefore, we merged the latter two groups and reassessed OS (Fig. 3H). Patients with high Ki-67 or EZH2+ MCL showed inferior OS compared to patients with low Ki-67/EZH2-negative MCL (*p* = 0.0121). Although the high Ki-67/EZH2+ MCL patient group showed the worst outcome, it was not statistically significant from patients with high Ki-67 MCL or EZH2+ MCL (*p* = 0.1262). In MCL patients with no p53 overexpression, EZH2 did not further stratify this group (*p* = 0.3793). EZH2 was not further assessed in MCL cases with p53 overexpression since all but one case was EZH2+. We also analyzed MCL cases for EED and SUZ12. Expression of these molecules in MCL did not correlate with either OS or RFS (Supplementary Fig. 3C–F).

### EZH2 expression correlated with gene expression profile

**Fig. 4:**
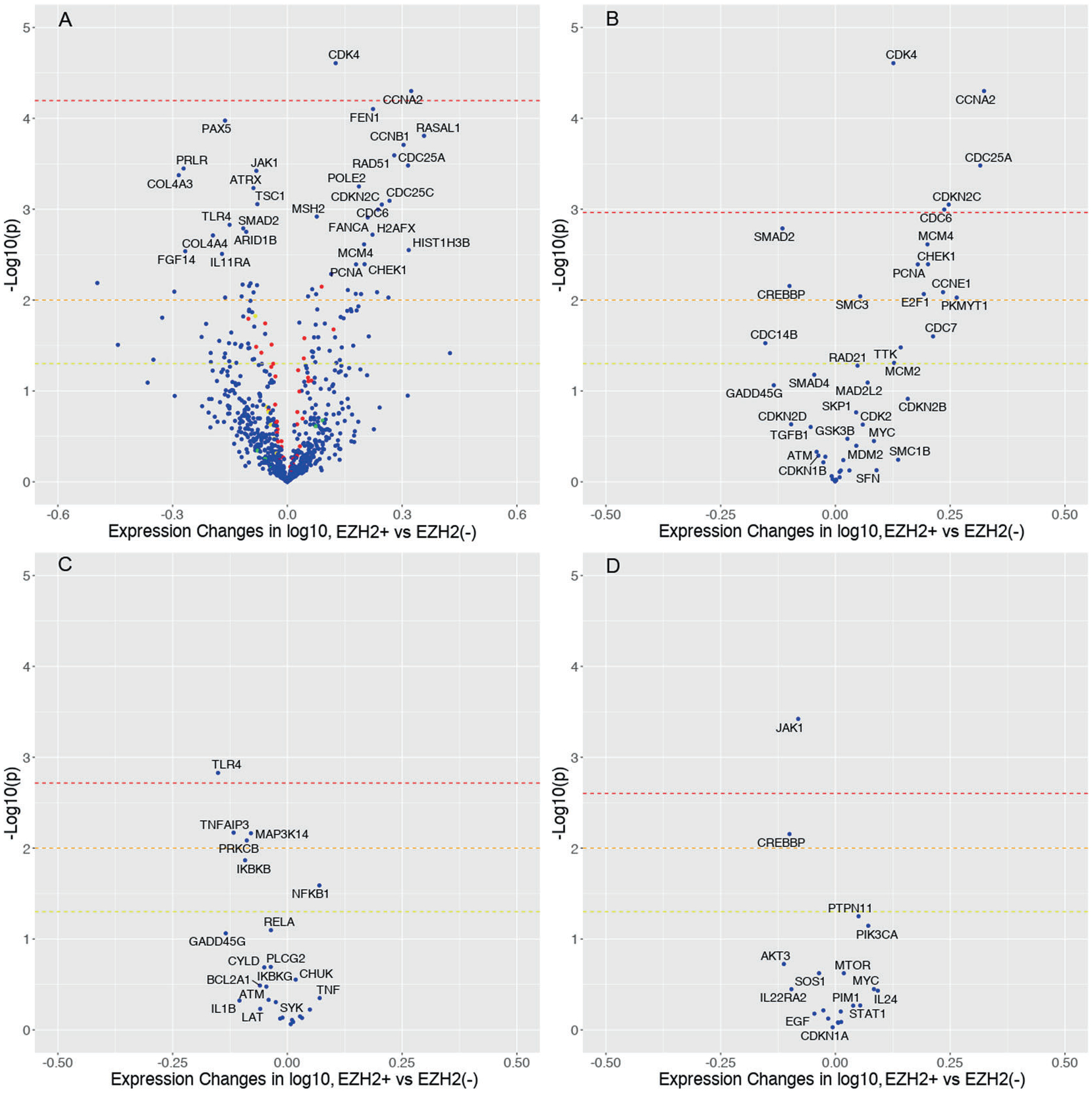
Volcano plot for differential expression between EZH2-overexpressed group and EZH2 not overexpressed group. **A** All the interrogated genes with top 30 genes with smallest *p* values labeled (Bonferroni-corrected *α* = 6.38 × 10^−5^). **B** Cell cycle pathway related genes (KEGG: hsa04110). **C** NF-κB pathway related genes (KEGG: hsa04064). **D** JAK-STAT pathway related genes (KEGG: hsa04630). Blue dots: interrogated genes; Red dots: housekeeping genes as control; yellow dots: positive controls (spiked in); green dots: negative controls (noise). Red dash line: Bonferroni-corrected cutoff of each gene set; orange dash line: *α* = 0.01; yellow dash line: (*α* = 0.05).

A total of 31 patients (12 and 10 patients for EZH2+ and EZH2(−) MCL, respectively) had gene expression profiles that passed quality control. Using the Pearson correlation test, a moderate correlation was observed between EZH2 expression determined by immunohistochemistry and *EZH2* mRNA expression (*r* = 0.553, *p* = 0.0005) (Supplementary Fig. 2H). The mean *EZH2* mRNA transcripts in EZH2+ and EZH2(−) MCL patients were 1217 (range: 809–2139) and 751 (range: 130–1556), respectively (*p* = 0.014). Table 2 shows differently expressed genes in EZH2+ MCL (*p* < 0.001). Particularly, *CDK4* and *CCNA2* were significantly overexpressed after Bonferroni correction with the total number of genes in the EZH2+ MCL group (Fig. 4A). The pathway enrichment analysis for the overexpressed genes in EZH2+ MCL revealed significant enrichment in cell cycle and mitotic transition pathways (Supplementary Table 1). On the other hand, this analysis for the underexpressed genes did not reveal meaningful pathways (Supplementary Table 2).

We further explored four common driving signaling pathways in lymphoid neoplasms. The Bonferroni corrections were performed with the numbers of genes included in each pathway gene set analysis. The cell cycle pathway (KEGG: hsa04110) had five significantly overexpressed genes (*CDK4*, *CCNA2*, *CDC25A*, *CDKN2C*, and *CDC6*) that passed the Bonferroni-corrected cutoff in the EZH2+ MCL (Fig. 4B). In contrast, *TLR4* (underexpression) and *JAK1* (underexpression) were the only significant genes after the Bonferroni correction in the NF-κB pathway (KEGG: hsa04064) and the JAK-STAT pathway (KEGG: hsa04630), respectively (Fig. 4C, D).

### Multivariate analysis

In univariate analysis, EZH2 expression, high (≥30%) Ki-67 proliferation rate, p53 overexpression (≥50%), and aggressive histologic variants showed increased hazard ratios for OS (Table 3 and Supplementary Fig. 4). In multivariate analysis, EZH2, p53, and aggressive histology failed to remain as independent predictors of OS. Only Ki-67 proliferation rate showed a marginal *p* value (*p* = 0.063).

## DISCUSSION

To the best of our knowledge, we present the largest cohort of MCL cases assessed for EZH2 expression. Earlier studies on this topic were performed in cell lines or included small cohorts of MCL cases^24–26,32–35^. In this study we assessed tumor specimens obtained from 166 untreated MCL patients. We used a cutoff of 40% to define EZH2 expression and we show that EZH2 expression is associated with a high proliferation rate, aggressive histologic variants, p53 overexpression, and poorer OS.

In line with our data, the correlation between EZH2 expression and Ki-67 proliferation rate has been observed in earlier studies. In the normal state, EZH2 expression is not present in the mantle zone of reactive follicles in lymph nodes. In a cell culture study, MCL cells did not express EZH2 in a resting state. When MCL cells were stimulated to proliferate, however, EZH2 expression was upregulated^26^. Another study also showed co-expression of EZH2 and Ki-67 in MCL cells using double immunofluorescence^24^.

Additionally, we found that EZH2 expression is significantly more common in aggressive variants of MCL, compared to cases with classic histology. The association of EZH2 with aggressive morphologic variants in other types of cancer has been reported, but not in MCL previously^36,37^. We showed that EZH2 expression can contribute to the identification of high-risk MCL patients, not only in patients with classic histology but with aggressive histology. We also show a correlation between p53 overexpression and EZH2 expression in MCL for the first time. A correlation between these biomarkers has been shown in oral squamous cell cancer and squamous cell carcinoma of the uterine cervix^38,39^. In breast cancer, a correlation between EZH2 expression and *TP53* mutation has been shown^40^. In a cell line study, EZH2 has been shown to bind the 5′-UTR of wild-type and mutated p53 mRNA and increases mRNA stability in a methyltransferase-independent manner^41^. It remains to be determined whether EZH2 expression is associated with *TP53* mutation in MCL. We also show that EZH2 expression is associated with poorer OS in MCL patients. However, EZH2 expression was not an independent risk factor for OS in multivariate analysis. Given that none of the analyzed parameters were statistically significant, the correlation of EZH2 with other parameters could be too interrelated to be analyzed separately. Nonetheless, our data suggest that EZH2 could be useful to identify high-risk MCL patients, particularly in combination with a high proliferate rate.

Demosthenous et al. reported that expression level of EZH2, EED, and SUZ12 proteins measured by Western blot in MCL cell lines was higher than normal CD19+ B cells and was comparable to FL cells lines irrespective of *EZH2* mutation status^35^. Using co-immunoprecipitation, they showed EED and SUZ12 from the precipitate captured by anti-EZH2 antibody, indicating active PRC2 function in MCL cell lines. Contrary to our expectations, EZH2 expression correlated weakly with expression of EED and SUZ12, other PRC2 complex components. The main difference between the study by Demosthenous et al. and ours is that they showed association of EZH2/EED/SUZ12 qualitatively and we demonstrate the correlation quantitatively using expression level. In this sense, our data is not contradictory to Demosthenous et al.’s study. Our data merely show a “weak” correlation between EZH2 and the other two PRC2 components (Supplementary Fig. 2C, D).

EZH2 expression showed no correlation with H3K27me3 by immunohistochemistry. Given that EZH2 is the main catalytic unit of PRC2, and that enhanced trimethylation at H3K27 has been shown in DLBCL cell lines harboring *EZH2* gain-of-function mutations^42,43^, our data suggest that EZH2 expression might have a PRC2-independent role in MCL. In addition, an inverse correlation between MHC-I or MHC-II expression and *EZH2* mutation is known in DLBCL^31^. In this MCL cohort, however, there was almost no loss of MHC-I or minimal loss in MHC-II molecules irrespective of EZH2 expression. Our data further support a non-canonical role of EZH2 in MCL. Indeed, accumulating data show that EZH2 transactivates downstream genes in a PRC2-independent manner. In glioblastoma multiforme, EZH2 binds to STAT3 enhancing STAT3 activity by increased tyrosine phosphorylation of STAT3^44^. EZH2 induces constitutive activation of NF-κB target gene expression via physical interaction with NF-κB subunits RelA/RelB in breast cancer^45^. In NK/T-cell lymphoma, EZH2 directly activates *CCND1* transcription independent of methyltransferase activity^46^.

Our GEP data show that *EZH2* expression is extensively associated with the overexpression of cell cycle related genes. For example, *CDK4* couples with cyclin D1 to activate the E2F1 transcription factor to transcribe cell cycle effectors such as *CCNA2* and *CCNE1*^47^. *CDC25A*, transcribed by activated E2F, removes the inhibitory phosphorylation in cyclin-dependent kinases (CDKs), such as CDK2, CDK4, and CDK6, and positively regulates the activities of CDKs that lead to cell cycle progression^48^. Interestingly, *CDKN2C*, which encodes CDK4/6 inhibitor, was also overexpressed in EZH2+ MCL (Table 2). Although *CDKN2C* negatively controls the cell cycle progression, its function could be overcome by overexpression of *CDK4, CCNA2, CCNB1, CDC25A*, and *CDC25C* whose function is to promote cell cycle and cell proliferation. The increased expressions of these genes suggest that EZH2 expression may potentiate the cell cycling machinery thus facilitating oncogenesis and behavior of CCND1 overexpression in MCL. In contrast, genes involved in NF-κB/B-cell receptor signaling pathway or JAK-STAT signaling pathway are not overexpressed in EZH2 + MCL. Therefore, our findings suggest that EZH2 expression in MCL is mostly associated with cyclin D1-related cell cycle machinery activation.

Intriguingly, our data is quite similar to those reported by Papakonstantinou et al. who assessed EZH2 expression in chronic lymphocytic leukemia (CLL)^49^. In their study, higher EZH2 mRNA expression is associated with unmutated CLL, a clinically aggressive subset of CLL, higher clinical stage and high ZAP 70 expression, which is another biomarker in CLL and is associated with worse clinical outcome. Correlation of EZH2 mRNA level and EZH2 protein level is also observed in CLL. Furthermore, EZH2-high CLL cases show shorter OS in univariate analysis, but does not retain independent significant in multivariate analysis, similar to our data.

An EZH2 inhibitor, tazemetostat, was approved by the Food and Drug Administration in June 2020 for adult patients with *EZH2*-mutated relapsed or refractory FL. The clinical trial on which the approval was based also showed durable response to FL patients with wild-type EZH2, suggesting application of the EZH2 inhibitor might not be limited to patients with *EZH2* mutation^12^. Indeed, MCL cell lines treated with EZH2 inhibitors (GSK343 or GSK126) showed reduced proliferation and decreased survival^35^. Taken together, our data identify MCL patients who might get benefited by EZH2 inhibitor.

In conclusion, using an immunohistochemical method we showed EZH2 expression in 38% of MCL cases. EZH2 expression is associated with a high proliferation rate, p53 overexpression, aggressive histologic variants and a poorer OS. Based on gene expression profiling data, EZH2 expression could potentiate cell cycle machinery in MCL. This activity also could be independent of the PRC2 complex because there was only weak or no correlation between EZH2 expression and expression of EED, SUZ12, H3K27me3, MHC-I, and MHC-II. These data also suggest that EZH2 could be useful to identify high-risk MCL patients.

## Supplementary Material

1

## Figures and Tables

**Table 1.** Clinicopathologic characteristics in EZH2-overexpressed mantle cell lymphoma.

	EZH2+, *N* (%)	EZH2(−), *N* (%)	*p*
Patients	57 (38)	93 (62)	NA
Gender			
Male	47	78	0.8214
Female	10	15	
Age, y			
Median (range)	61 (42 to 87)	60 (39 to 88)	0.347
<60	28	47	0.8664
≥60	29	46	
Site			0.6317
Lymph node	44	69	
Spleen	3	2	
Tonsil	4	8	
Extranodal site	6	14	
Stage			0.7675
I/II	5 (9)	7 (8)	
III/IV	50 (91)	82 (92)	
MIPI			0.2839
Low	18 (42)	38 (50)	
Intermediate	13 (30)	26 (34)	
High	12 (28)	12 (16)	
Pathology			**<0.0001**
Classic	20 (35)	66 (71)	
Aggressive	37 (65)	27 (29)	
Pattern			0.2826
Mantle zone	0 (0)	2 (2)	
Diffuse	33 (58)	44 (47)	
Nodular	24 (42)	47 (51)	
Biomarkers			
SOX11 +	48/49 (98)	64/67 (96)	0.4774
Ki-67 high	41/57 (72)	17/91 (19)	**<0.0001**
P53 overexpression	15/35 (43)	1/48 (2)	**<0.0001**
CD5+	57/57 (100)	86/91 (95)	0.1568
CD10+	2/38 (5)	0/71 (0)	0.1194
MHC-I loss	1/40 (3)	0/56 (0)	0.1734
MHC-II loss	3/36 (8)	4/50 (8)	>0.9999

Statistically significant *p* < 0.05 values are in bold.

**Table 2.** Differentially expressed genes in EZH2-positive mantle cell lymphoma.

Overexpressed	*p* value (two tailed)
*CDK4*	2.47169E–05
*CCNA2*	5.01135E–05
*FEN1*	7.90567E-05
*RASAL1*	0.000155936
*CCNB1*	0.000195885
*RAD51*	0.000255449
*CDC25A*	0.000330412
*POLE2*	0.000561942
*CDC25C*	0.000808507
*CDKN2C*	0.000889433
Underexpressed	*p* value (two tailed)
*PAX5*	0.000105915
*PRLR*	0.000356705
*JAK1*	0.000378505
*COL4A3*	0.000422591
*ATRX*	0.000585516
*TSC1*	0.00088001

**Table 3.** Univariate and multivariate analysis.

		Univariate			Multivariate	
	HR	95% CI	*p* value	HR	95% CI	*p* value
EZH2+	2.037	1.306–3.178	0.002	1.559	0.668–3.636	0.305
Ki-67 high	2.47	1.616–3.773	<0.001	2.315	0.960–4.749	0.063
P53 overexpression	1.947	1.058–3.584	0.032	1.41	0.613–3.244	0.419
Aggressive cytology	1.564	1.027–2.381	0.037	0.619	0.276–1.389	0.245

## Data Availability

All data generated or analyzed during this study are included in this published article.

## References

[1] Swerdlow SH The 2016 revision of the World Health Organization classification of lymphoid neoplasms. Blood 127, 2375–2390 (2016). 26980727 10.1182/blood-2016-01-643569 PMC4874220

[2] Geisler CH The Mantle Cell Lymphoma International Prognostic Index (MIPI) is superior to the International Prognostic Index (IPI) in predicting survival following intensive first-line immunochemotherapy and autologous stem cell transplantation (ASCT). Blood 115, 1530–1533 (2010). 20032504 10.1182/blood-2009-08-236570

[3] Geisler CH Nordic MCL2 trial update: six-year follow-up after intensive immunochemotherapy for untreated mantle cell lymphoma followed by BEAM or BEAC+ autologous stem-cell support: still very long survival but late relapses do occur. Br. J. Haematol 158, 355–362 (2012). 22640180 10.1111/j.1365-2141.2012.09174.x

[4] Romaguera JE Ten-year follow-up after intense chemoimmunotherapy with Rituximab-HyperCVAD alternating with Rituximab-high dose methotrexate/cytarabine (R-MA) and without stem cell transplantation in patients with untreated aggressive mantle cell lymphoma. Br. J. Haematol 150, 200–208 (2010). 20528872 10.1111/j.1365-2141.2010.08228.x

[5] Cao Q Coordinated regulation of polycomb group complexes through microRNAs in cancer. Cancer Cell 20, 187–199 (2011). 21840484 10.1016/j.ccr.2011.06.016 PMC3157014

[6] Ezponda T & Licht JD Molecular pathways: deregulation of histone h3 lysine 27 methylation in cancer-different paths, same destination. Clin. Cancer Res 20, 5001–5008 (2014). 24987060 10.1158/1078-0432.CCR-13-2499 PMC4184969

[7] Pasini D Characterization of an antagonistic switch between histone H3 lysine 27 methylation and acetylation in the transcriptional regulation of Polycomb group target genes. Nucleic Acids Res 38, 4958–4969 (2010). 20385584 10.1093/nar/gkq244 PMC2926606

[8] Varambally S The polycomb group protein EZH2 is involved in progression of prostate cancer. Nature 419, 624–629 (2002). 12374981 10.1038/nature01075

[9] Kleer CG EZH2 is a marker of aggressive breast cancer and promotes neoplastic transformation of breast epithelial cells. Proc. Natl Acad. Sci. U.S.A 100, 11606–11611 (2003). 14500907 10.1073/pnas.1933744100 PMC208805

[10] Bodor C EZH2 mutations are frequent and represent an early event in follicular lymphoma. Blood 122, 3165–3168 (2013). 24052547 10.1182/blood-2013-04-496893 PMC3814734

[11] Morin RD Somatic mutations altering EZH2 (Tyr641) in follicular and diffuse large B-cell lymphomas of germinal-center origin. Nat. Genet 42, 181–185 (2010). 20081860 10.1038/ng.518 PMC2850970

[12] Morschhauser F Tazemetostat for patients with relapsed or refractory follicular lymphoma: an open-label, single-arm, multicentre, phase 2 trial. Lancet Oncol 21, 1433–1442 (2020). 33035457 10.1016/S1470-2045(20)30441-1 PMC8427481

[13] Bea S Landscape of somatic mutations and clonal evolution in mantle cell lymphoma. Proc. Natl Acad. Sci. U.S.A 110, 18250–18255 (2013). 24145436 10.1073/pnas.1314608110 PMC3831489

[14] Wu C Genetic heterogeneity in primary and relapsed mantle cell lymphomas: Impact of recurrent CARD11 mutations. Oncotarget 7, 38180–38190 (2016). 27224912 10.18632/oncotarget.9500 PMC5122381

[15] Zhang J The genomic landscape of mantle cell lymphoma is related to the epigenetically determined chromatin state of normal B cells. Blood 123, 2988–2996 (2014). 24682267 10.1182/blood-2013-07-517177 PMC4014841

[16] Meissner B The E3 ubiquitin ligase UBR5 is recurrently mutated in mantle cell lymphoma. Blood 121, 3161–3164 (2013). 23407552 10.1182/blood-2013-01-478834

[17] Kridel R Whole transcriptome sequencing reveals recurrent NOTCH1 mutations in mantle cell lymphoma. Blood 119, 1963–1971 (2012). 22210878 10.1182/blood-2011-11-391474

[18] Zhao S Efficacy of venetoclax in high risk relapsed mantle cell lymphoma (MCL) - outcomes and mutation profile from venetoclax resistant MCL patients. Am. J. Hematol 95, 623–629 (2020). 32239765 10.1002/ajh.25796

[19] Jain P Long-term outcomes and mutation profiling of patients with mantle cell lymphoma (MCL) who discontinued ibrutinib. Br. J. Haematol 183, 578–587 (2018). 30175400 10.1111/bjh.15567

[20] Jain P Genomic profiles and clinical outcomes of de novo blastoid/pleomorphic MCL are distinct from those of transformed MCL. Blood Adv 4, 1038–1050 (2020). 32191807 10.1182/bloodadvances.2019001396 PMC7094021

[21] Pararajalingam P Coding and noncoding drivers of mantle cell lymphoma identified through exome and genome sequencing. Blood 136, 572–584 (2020). 32160292 10.1182/blood.2019002385 PMC7440974

[22] Nadeu F Genomic and epigenomic insights into the origin, pathogenesis, and clinical behavior of mantle cell lymphoma subtypes. Blood 136, 1419–1432 (2020). 32584970 10.1182/blood.2020005289 PMC7498364

[23] Kim DH Mantle cell lymphoma involving skin: a clinicopathologic study of 37 cases. Am. J. Surg. Pathol 43, 1421–1428 (2019). 31219818 10.1097/PAS.0000000000001312

[24] van Kemenade FJ Coexpression of BMI-1 and EZH2 polycomb-group proteins is associated with cycling cells and degree of malignancy in B-cell non-Hodgkin lymphoma. Blood 97, 3896–3901 (2001). 11389032 10.1182/blood.v97.12.3896

[25] Abd Al Kader L In aggressive variants of non-Hodgkin lymphomas, Ezh2 is strongly expressed and polycomb repressive complex PRC1.4 dominates over PRC1.2. Virchows Arch 463, 697–711 (2013). 23948956 10.1007/s00428-013-1428-y

[26] Visser HP The Polycomb group protein EZH2 is upregulated in proliferating, cultured human mantle cell lymphoma. Br. J. Haematol 112, 950–958 (2001). 11298590 10.1046/j.1365-2141.2001.02641.x

[27] Hoster E A new prognostic index (MIPI) for patients with advanced-stage mantle cell lymphoma. Blood 111, 558–565 (2008). 17962512 10.1182/blood-2007-06-095331

[28] World Medical A World Medical Association Declaration of Helsinki: ethical principles for medical research involving human subjects. JAMA 310, 2191–2194 (2013). 24141714 10.1001/jama.2013.281053

[29] Hoster E Prognostic value of Ki-67 index, cytology, and growth pattern in mantle-cell lymphoma: results from randomized trials of the European mantle cell lymphoma network. J. Clin. Oncol 34, 1386–1394 (2016). 26926679 10.1200/JCO.2015.63.8387

[30] Aukema SM Expression of TP53 is associated with the outcome of MCL independent of MIPI and Ki-67 in trials of the European MCL Network. Blood 131, 417–420 (2018). 29196411 10.1182/blood-2017-07-797019

[31] Ennishi D Molecular and genetic characterization of MHC deficiency identifies EZH2 as therapeutic target for enhancing immune recognition. Cancer Discov 9, 546–563 (2019). 30705065 10.1158/2159-8290.CD-18-1090

[32] Tian X, Pelton A, Shahsafaei A & Dorfman DM Differential expression of enhancer of zeste homolog 2 (EZH2) protein in small cell and aggressive B-cell non-Hodgkin lymphomas and differential regulation of EZH2 expression by p-ERK1/2 and MYC in aggressive B-cell lymphomas. Mod. Pathol 29, 1050–1057 (2016). 27282353 10.1038/modpathol.2016.114

[33] Romanchikova N & Trapencieris P Wedelolactone targets EZH2-mediated Histone H3K27 methylation in mantle cell lymphoma. Anticancer Res 39, 4179–4184 (2019). 31366503 10.21873/anticanres.13577

[34] Kanduri M A key role for EZH2 in epigenetic silencing of HOX genes in mantle cell lymphoma. Epigenetics 8, 1280–1288 (2013). 24107828 10.4161/epi.26546 PMC3933489

[35] Demosthenous C Deregulation of polycomb repressive complex-2 in mantle cell lymphoma confers growth advantage by epigenetic suppression of cdkn2b. Front. Oncol 10, 1226 (2020). 32850364 10.3389/fonc.2020.01226 PMC7396700

[36] Rao RC, Chan MP, Andrews CA & Kahana A EZH2, proliferation rate, and aggressive tumor subtypes in cutaneous basal cell carcinoma. JAMA Oncol 2, 962–963 (2016). 27054919 10.1001/jamaoncol.2016.0021 PMC4945394

[37] Bachmann IM EZH2 expression is associated with high proliferation rate and aggressive tumor subgroups in cutaneous melanoma and cancers of the endometrium, prostate, and breast. J. Clin. Oncol 24, 268–273 (2006). 16330673 10.1200/JCO.2005.01.5180

[38] Shiogama S, Yoshiba S, Soga D, Motohashi H & Shintani S Aberrant expression of EZH2 is associated with pathological findings and P53 alteration. Anticancer Res 33, 4309–4317 (2013). 24122997

[39] Zhang HM, Chen SQ & Yao SZ Expression and clinical implications of enhancer of Zeste homolog 2 and p53 protein in squamous cell carcinoma and precancerous lesions in the cervix. Genet. Mol. Res 15, gmr.15027408 (2016). 10.4238/gmr.15027408 27323178

[40] Pietersen AM EZH2 and BMI1 inversely correlate with prognosis and TP53 mutation in breast cancer. Breast Cancer Res 10, R109 (2008). 19099573 10.1186/bcr2214 PMC2656906

[41] Zhao Y EZH2 cooperates with gain-of-function p53 mutants to promote cancer growth and metastasis. EMBO J 38, e99599 (2019). 30723117 10.15252/embj.201899599 PMC6396169

[42] McCabe MT Mutation of A677 in histone methyltransferase EZH2 in human B-cell lymphoma promotes hypertrimethylation of histone H3 on lysine 27 (H3K27). Proc. Natl Acad. Sci. U.S.A 109, 2989–2994 (2012). 22323599 10.1073/pnas.1116418109 PMC3287005

[43] Yap DB Somatic mutations at EZH2 Y641 act dominantly through a mechanism of selectively altered PRC2 catalytic activity, to increase H3K27 trimethylation. Blood 117, 2451–2459 (2011). 21190999 10.1182/blood-2010-11-321208 PMC3062411

[44] Kim E Phosphorylation of EZH2 activates STAT3 signaling via STAT3 methylation and promotes tumorigenicity of glioblastoma stem-like cells. Cancer Cell 23, 839–852 (2013). 23684459 10.1016/j.ccr.2013.04.008 PMC4109796

[45] Lee ST Context-specific regulation of NF-kappaB target gene expression by EZH2 in breast cancers. Mol. Cell 43, 798–810 (2011). 21884980 10.1016/j.molcel.2011.08.011

[46] Yan J EZH2 overexpression in natural killer/T-cell lymphoma confers growth advantage independently of histone methyltransferase activity. Blood 121, 4512–4520 (2013). 23529930 10.1182/blood-2012-08-450494

[47] Mok MT CCRK is a novel signalling hub exploitable in cancer immunotherapy. Pharmacol. Ther 186, 138–151 (2018). 29360538 10.1016/j.pharmthera.2018.01.008

[48] Shen T & Huang S The role of Cdc25A in the regulation of cell proliferation and apoptosis. Anticancer Agents Med. Chem 12, 631–639 (2012). 22263797 10.2174/187152012800617678 PMC3544488

[49] Papakonstantinou N The histone methyltransferase EZH2 as a novel pro-survival factor in clinically aggressive chronic lymphocytic leukemia. Oncotarget 7, 35946–35959 (2016). 27191993 10.18632/oncotarget.9371 PMC5094974

