# Supplementary material for: EZH2 expression is associated with inferior overall survival in mantle cell lymphoma
Source: Mod Pathol. Author manuscript; Available in PMC 2023 Oct 10. (PMC10563799; doi:10.1038/s41379-021-00885-9)
Supplement: 1 [file NIHMS1936015-supplement-1.pdf]

Supplementary figures and tables

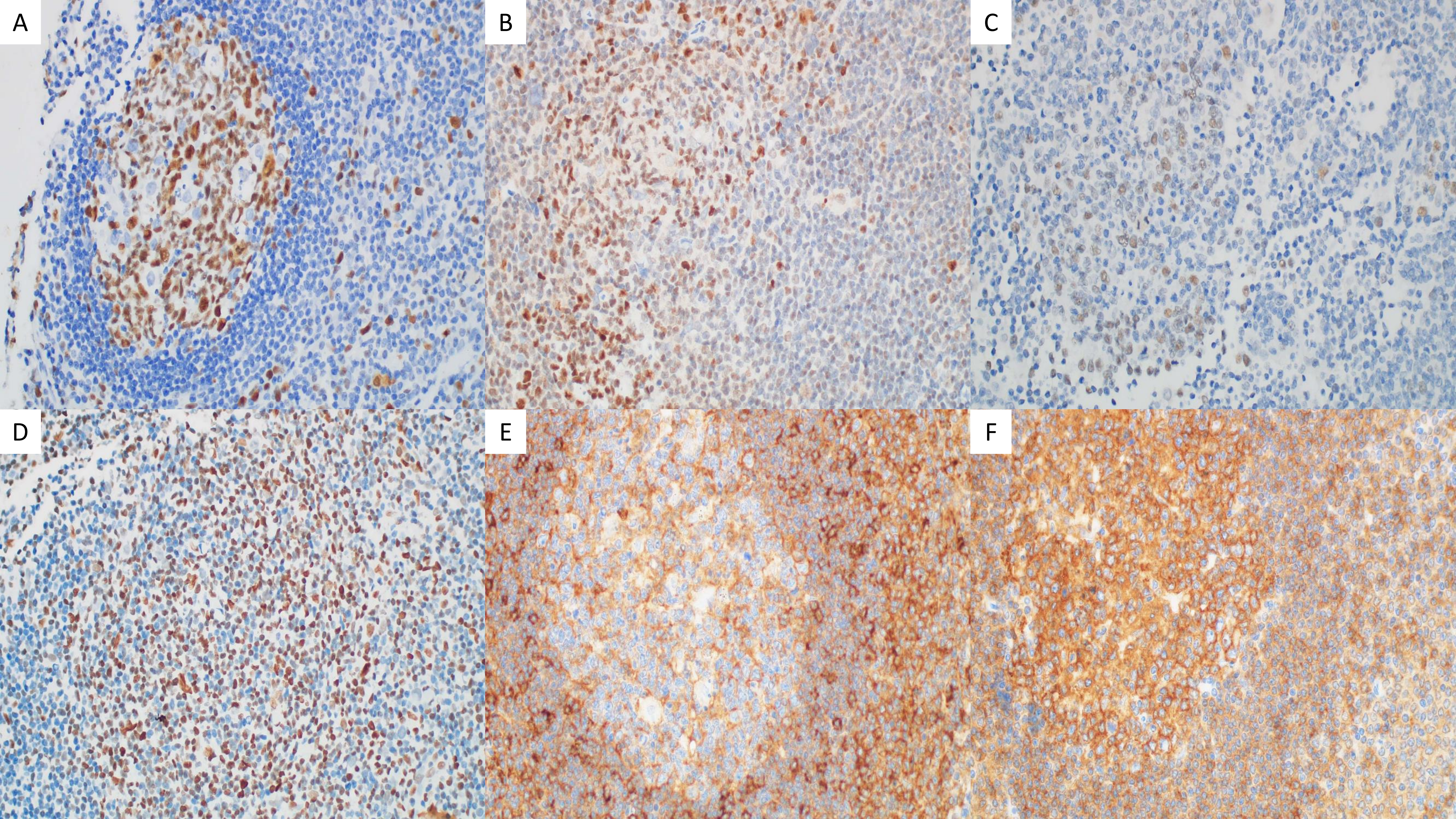
A
B
C
D
E
F

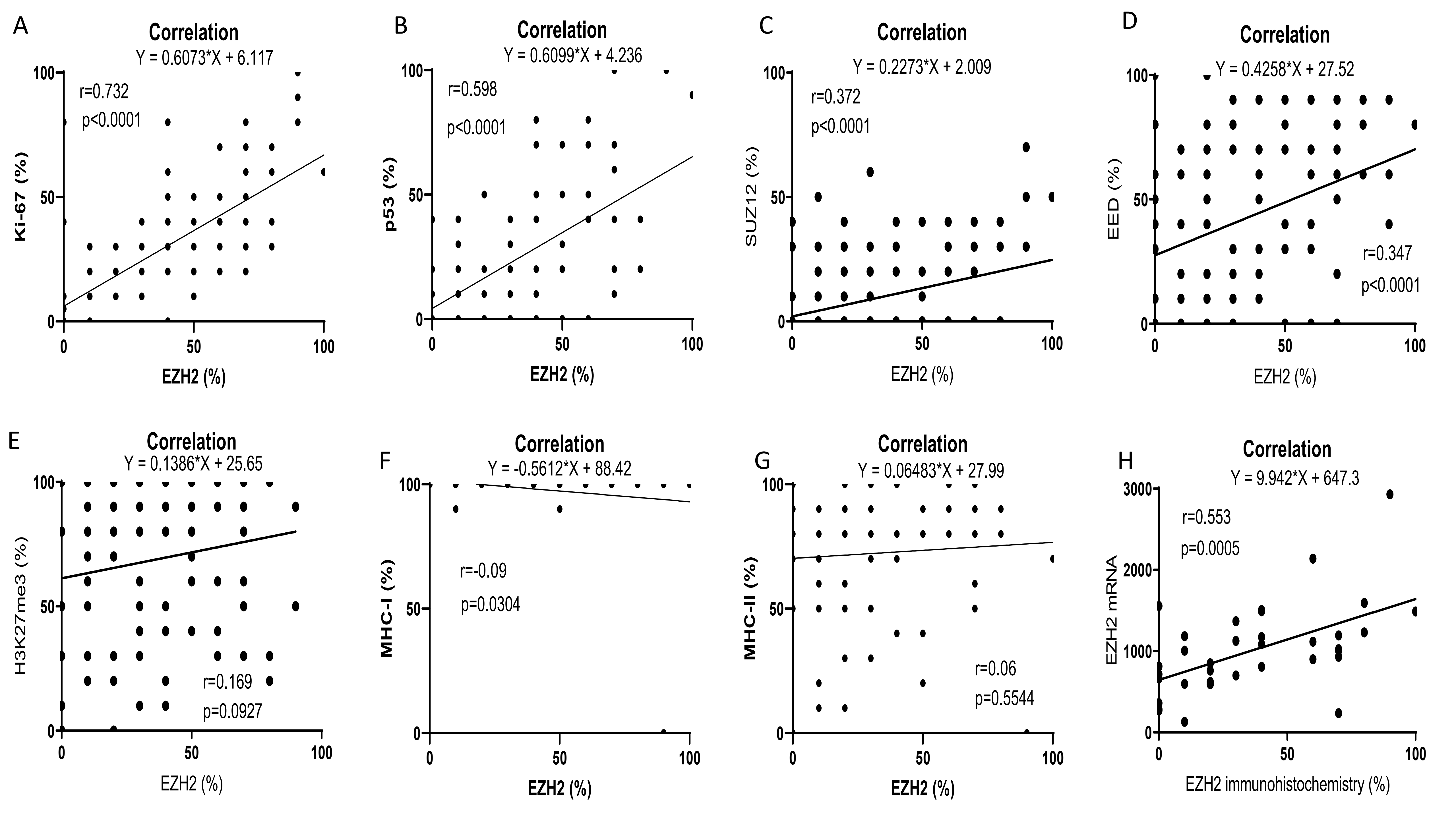
A
Correlation
Y = 0.6073*X + 6.117
r=0.732
p<0.0001
Ki-67 (%)
EZH2 (%)
B
Correlation
Y = 0.6099*X + 4.236
r=0.598
p<0.0001
p53 (%)
EZH2 (%)
C
Correlation
Y = 0.2273*X + 2.009
r=0.372
p<0.0001
SUZ12 (%)
EZH2 (%)
D
Correlation
Y = 0.4258*X + 27.52
r=0.347
p<0.0001
EED (%)
EZH2 (%)
E
Correlation
Y = 0.1386*X + 25.65
r=0.169
p=0.0927
H3K27me3 (%)
EZH2 (%)
F
Correlation
Y = -0.5612*X + 88.42
r=-0.09
p=0.0304
MHC-I (%)
EZH2 (%)
G
Correlation
Y = 0.06483*X + 27.99
r=0.06
p=0.5544
MHC-II (%)
EZH2 (%)
H
Correlation
Y = 9.942*X + 647.3
r=0.553
p=0.0005
EZH2 mRNA
EZH2 immunohistochemistry (%)

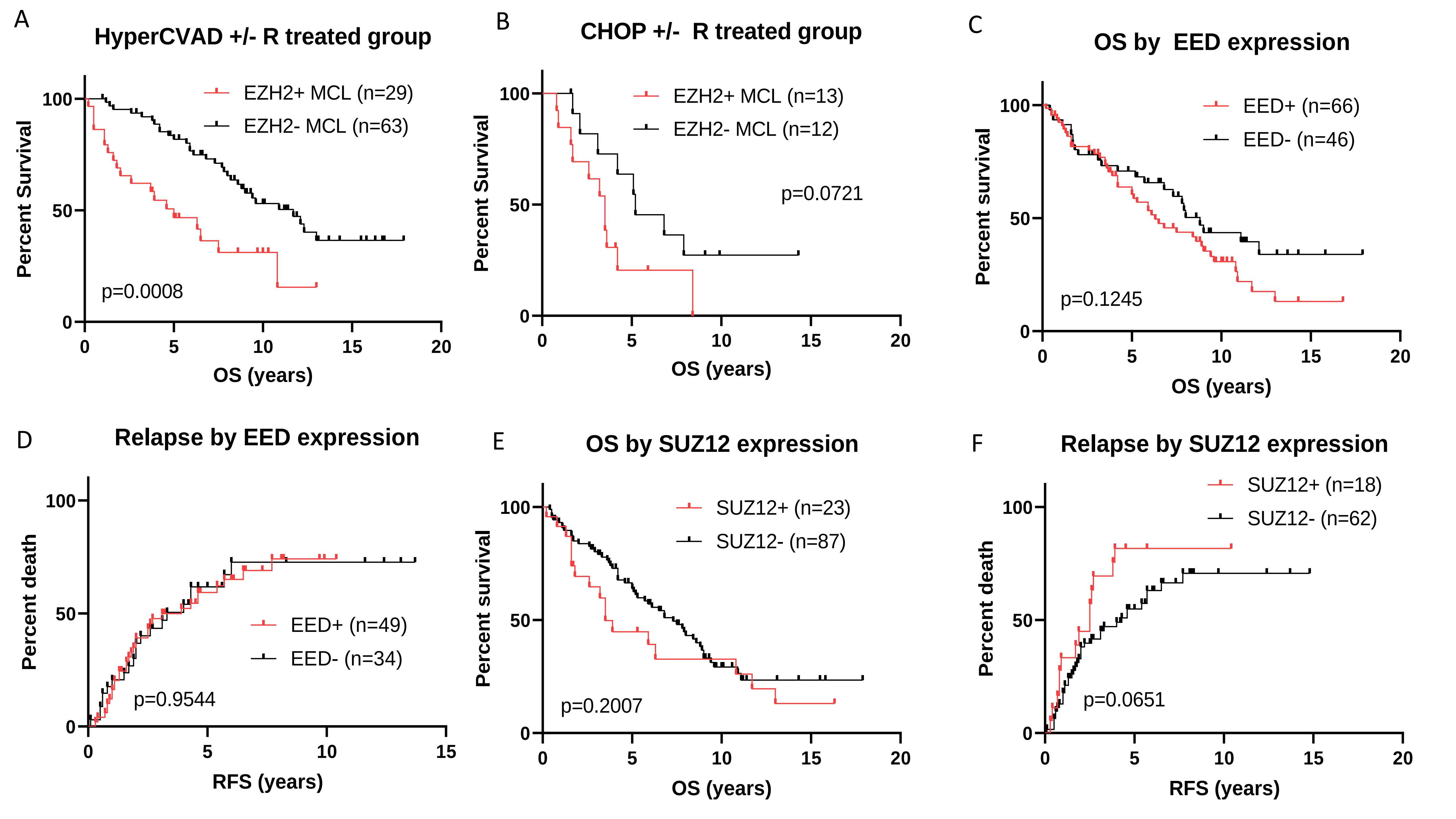
A
HyperCVAD +/- R treated group
EZH2+ MCL (n=29)
EZH2- MCL (n=63)
p=0.0008
Percent Survival
OS (years)
B
CHOP +/- R treated group
EZH2+ MCL (n=13)
EZH2- MCL (n=12)
p=0.0721
Percent Survival
OS (years)
C
OS by EED expression
EED+ (n=66)
EED- (n=46)
p=0.1245
Percent survival
OS (years)
D
Relapse by EED expression
EED+ (n=49)
EED- (n=34)
p=0.9544
Percent death
RFS (years)
E
OS by SUZ12 expression
SUZ12+ (n=23)
SUZ12- (n=87)
p=0.2007
Percent survival
OS (years)
F
Relapse by SUZ12 expression
SUZ12+ (n=18)
SUZ12- (n=62)
p=0.0651
Percent death
RFS (years)

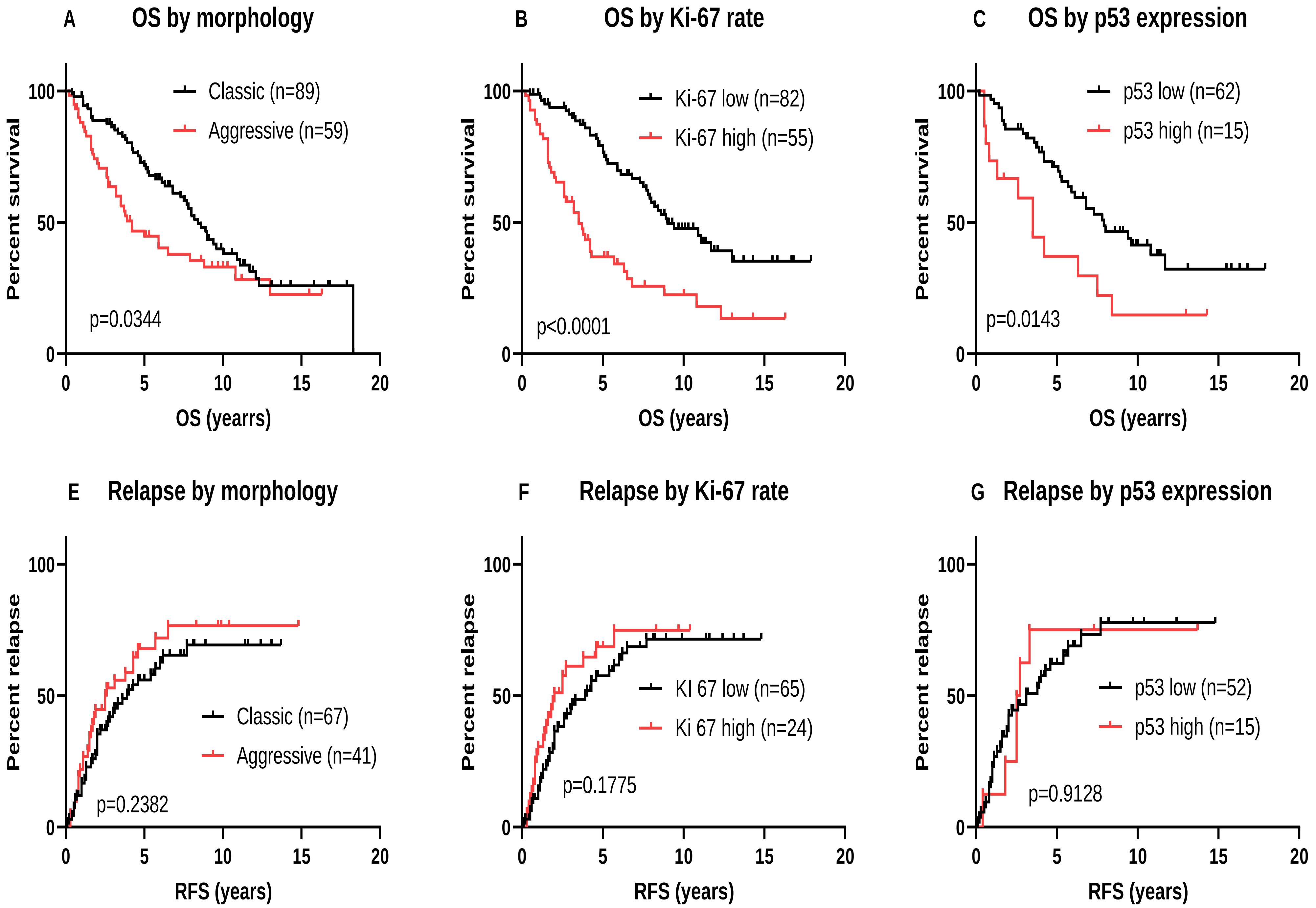
A
OS by morphology
Classic (n=89)
Aggressive (n=59)
Percent survival
p=0.0344
OS (yearrs)
B
OS by Ki-67 rate
Ki-67 low (n=82)
Ki-67 high (n=55)
Percent survival
p<0.0001
OS (years)
C
OS by p53 expression
p53 low (n=62)
p53 high (n=15)
Percent survival
p=0.0143
OS (yearrs)
E
Relapse by morphology
Percent relapse
Classic (n=67)
Aggressive (n=41)
p=0.2382
RFS (years)
F
Relapse by Ki-67 rate
Percent relapse
KI 67 low (n=65)
Ki 67 high (n=24)
p=0.1775
RFS (years)
G
Relapse by p53 expression
Percent relapse
p53 low (n=52)
p53 high (n=15)
p=0.9128
RFS (years)

Supplementary figure legend

S figure 1. Expression pattern of each marker in normal germinal centers. A. EZH2 expression in normal germinal centers (x40). B. EED expression in normal germinal centers (x40). C. SUZ12 expression in normal germinal centers (x40). D. H3K27me3 expression in normal germinal centers (x40). E. MHC-I expression in normal germinal centers (x40). F. MHC-II expression in normal germinal centers (x40).

S figure 2. Correlation between EZH2 and various markers. r: Pearson's correlation.

S figure 3. Overall survival (OS) regarding EZH2 expression in patients treated with hyperCVAD ± rituximab (S figure 3-A) and in patients treated with CHOP ± rituximab (S figure 3-B). OS and relapse-free survival (RFS) regarding EED (S figure 3-C and -D) and SUZ12 expression (S figure 3-E and -F).

HyperCVAD: cyclophosphamide, vincristine sulfate, doxorubicin hydrochloride, and dexamethasone

CHOP: cyclophosphamide, doxorubicin hydrochloride, vincristine sulfate, and prednisone

S figure 4. Overall survival (OS) and relapse-free survival (RFS) regarding morphology (S figure 4-A and -E), Ki-67 rate (S figure 4-B and -D) and p53 expression (S figure 4-C and -G).

Supplement Table 1. The pathway enrichment analysis for the overexpressed genes in mantle cell lymphoma with EZH2 expression.

Pathway ID	p-value	FDRs	Pathway name	Gene name	-log(p-value)
R-HSA-453279	1.11022E-16	1.15463E-14	Mitotic G1 phase and G1/S transition	CCNA2, CCNB1, CDC25A, CDC6, CDK4, CDKN2C, MCM4, PCNA, POLE2	15.95458977
R-HSA-1640170	1.11022E-16	1.15463E-14	Cell Cycle	CCNA2, CCNB1, CDC25A, CDC25C, CDC6, CDK4, CDKN2C, CHEK1, FEN1, H2AFX, MCM4, PCNA, POLE2, RAD51	15.95458977
R-HSA-69278	1.11022E-16	1.15463E-14	Cell Cycle, Mitotic	CCNA2, CCNB1, CDC25A, CDC25C, CDC6, CDK4, CDKN2C, FEN1, H2AFX, MCM4, PCNA, POLE2	15.95458977
R-HSA-69206	5.66214E-15	4.41647E-13	G1/S Transition	CCNA2, CCNB1, CDC25A, CDC6, CDK4, MCM4, PCNA, POLE2	14.24701959
R-HSA-1538133	1.09912E-14	6.81455E-13	G0 and Early G1	CCNA2, CDC25A, CDC6, PCNA	13.95895458
R-HSA-1362277	1.3755E-11	7.1526E-10	Transcription of E2F targets under negative control by DREAM complex	CDC25A, CDC6, PCNA	10.8615395
R-HSA-69242	6.93049E-11	3.04942E-09	S Phase	CCNA2, CDC25A, CDC6, CDK4, FEN1, MCM4, PCNA, POLE2	10.15923609
R-HSA-156711	1.28424E-09	5.00854E-08	Polo-like kinase mediated events	CCNB1, CDC25A, CDC25C	8.891353341
R-HSA-5693538	7.13511E-09	2.42594E-07	Homology Directed Repair	CCNA2, CHEK1, FEN1, H2AFX, PCNA, POLE2, RAD51	8.146599418
R-HSA-176187	1.75991E-08	5.45573E-07	Activation of ATR in response to replication stress	CDC25A, CDC25C, CDC6, CHEK1, MCM4	7.75450872
R-HSA-69481	2.26004E-08	6.3281E-07	G2/M Checkpoints	CCNB1, CDC25A, CDC25C, CDC6, CHEK1, H2AFX, MCM4	7.645884704

R-HSA-69205	2.85135E-08	7.41351E-07	G1/S-Specific Transcription	CDC25A, CDC6, PCNA	7.54494971
R-HSA-73894	3.46539E-08	8.31693E-07	DNA Repair	CCNA2, CHEK1, FANCA, FEN1, H2AFX, MSH2, PCNA, POLE2, RAD51	7.460247969
R-HSA-5693532	4.07635E-08	8.96798E-07	DNA Double-Strand Break Repair	CCNA2, CHEK1, FEN1, H2AFX, PCNA, POLE2, RAD51	7.389728102
R-HSA-69620	6.896E-08	1.3792E-06	Cell Cycle Checkpoints	CCNA2, CCNB1, CDC25A, CDC25C, CDC6, CHEK1, H2AFX, MCM4	7.161402898
R-HSA-6804114	1.10172E-07	2.09327E-06	TP53 Regulates Transcription of Genes Involved in G2 Cell Cycle Arrest	CCNB1, CDC25C, PCNA	6.957928273
R-HSA-5693567	1.78293E-07	3.20927E-06	HDR through Homologous Recombination (HRR) or Single Strand Annealing (SSA)	CCNA2, CHEK1, H2AFX, PCNA, POLE2, RAD51	6.74886607
R-HSA-6791312	2.18143E-07	3.70842E-06	TP53 Regulates Transcription of Cell Cycle Genes	CCNA2, CCNB1, CDC25C, PCNA	6.661259465
R-HSA-69239	2.68059E-07	4.02089E-06	Synthesis of DNA	CCNA2, CDC6, FEN1, MCM4, PCNA, POLE2	6.571769201
R-HSA-3700989	3.5452E-07	4.96328E-06	Transcriptional Regulation by TP53	CCNA2, CCNB1, CDC25C, CHEK1, MSH2, PCNA	6.450359429
R-HSA-69306	3.92073E-07	5.48902E-06	DNA Replication	CCNA2, CDC6, FEN1, MCM4, PCNA, POLE2	6.406633271
R-HSA-69273	5.84851E-07	7.60306E-06	Cyclin A/B1/B2 associated events during G2/M transition	CCNA2, CCNB1, CDC25A, CDC25C	6.232955054
R-HSA-69275	3.93503E-06	4.65065E-05	G2/M Transition	CCNA2, CCNB1, CDC25A, CDC25C	5.405051861

R-HSA-453274	4.1514E-06	4.65065E-05	Mitotic G2-G2/M phases	CCNA2, CCNB1, CDC25A, CDC25C	5.381805731
R-HSA-75035	4.20755E-06	4.65065E-05	Chk1/Chk2(Cds1) mediated inactivation of Cyclin B:Cdk1 complex	CCNB1, CDC25C, CHEK1	5.375970741
R-HSA-212436	4.22786E-06	4.65065E-05	Generic Transcription Pathway	CCNA2, CCNB1, CDC25C, CDK4, CHEK1, H2AFX, MSH2, PCNA, RAD51	5.373878965

FDR: false discovery rate

Supplement Table 2. The pathway enrichment analysis for the underexpressed genes in mantle cell lymphoma with EZH2 expression.

Pathway ID	p-value	FDRs	Pathway name	Gene name	-log(p-value)
R-HSA-8939246	5.49335E-07	0.000156011	RUNX1 regulates transcription of genes involved in differentiation of myeloid cells	CREBBP, PRKCB	6.260163078
R-HSA-8878171	1.3403E-06	0.000190322	Transcriptional regulation by RUNX1	ARID1B, CREBBP, PAX5, PRKCB, SMARCB1	5.872799209
R-HSA-2214320	0.000215241	0.019595269	Anchoring fibril formation	COL4A3, COL4A4	3.667075747
R-HSA-6788467	0.00027599	0.019595269	IL-6-type cytokine receptor ligand interactions	IL11RA, JAK1	3.559107118
R-HSA-2243919	0.00054677	0.025389374	Crosslinking of collagen fibrils	COL4A3, COL4A4	3.262195237
R-HSA-912526	0.0007949	0.025389374	Interleukin receptor SHC signaling	IL5RA, JAK1	3.099687337
R-HSA-212436	0.000881397	0.025389374	Generic Transcription Pathway	ARID1B, CREBBP, PAX5, PRKCB, SMAD2, SMARCB1, TSC1	3.054828238
R-HSA-6783589	0.000906763	0.025389374	Interleukin-6 family signaling	IL11RA, JAK1	3.042506039
R-HSA-3000157	0.000906763	0.025389374	Laminin interactions	COL4A3, COL4A4	3.042506039
R-HSA-8939243	0.001425333	0.031341136	RUNX1 interacts with co-factors whose precise effect on RUNX1 targets is not known	ARID1B, SMARCB1	2.846083722
R-HSA-73857	0.001553548	0.031341136	RNA Polymerase II Transcription	ARID1B, CREBBP, PAX5, PRKCB, SMAD2, SMARCB1, TSC1	2.808675285
R-HSA-419037	0.00180646	0.031341136	NCAM1 interactions	COL4A3, COL4A4	2.743171571
R-HSA-8948216	0.00180646	0.031341136	Collagen chain trimerization	COL4A3, COL4A4	2.743171571
R-HSA-168256	0.002164191	0.031341136	Immune System	CREBBP, IL11RA, IL5RA, JAK1, MAP3K14, PPP3CB, PRKCB, PRLR, TLR4, TNFAIP3	2.664704391
R-HSA-512988	0.002320743	0.031341136	Interleukin-3, Interleukin-5 and GM-CSF signaling	IL5RA, JAK1	2.634372981
R-HSA-451927	0.002505812	0.031341136	Interleukin-2 family signaling	IL5RA, JAK1	2.601051483
R-HSA-3214858	0.002600884	0.031341136	RMTs methylate histone arginines	ARID1B, SMARCB1	2.584878989

R-HSA-74160	0.002769155	0.031341136	Gene expression (Transcription)	ARID1B, CREBBP, PAX5, PRKCB, SMAD2, SMARCB1, TSC1	2.55765272
R-HSA-9673013	0.002849194	0.031341136	Diseases of Telomere Maintenance	ATRX	2.545277949
R-HSA-9006821	0.002849194	0.031341136	Alternative Lengthening of Telomeres (ALT)	ATRX	2.545277949
R-HSA-9670621	0.002849194	0.031341136	Defective Inhibition of DNA Recombination at Telomere	ATRX	2.545277949
R-HSA-9670615	0.002849194	0.031341136	Defective Inhibition of DNA Recombination at Telomere Due to ATRX Mutations	ATRX	2.545277949
R-HSA-9670613	0.002849194	0.031341136	Defective Inhibition of DNA Recombination at Telomere Due to DAXX Mutations	ATRX	2.545277949
R-HSA-5621481	0.002895657	0.031852229	C-type lectin receptors (CLRs)	CREBBP, MAP3K14, PPP3CB	2.538252852
R-HSA-3000171	0.003421758	0.034217579	Non-integrin membrane-ECM interactions	COL4A3, COL4A4	2.465750725
R-HSA-2022090	0.004106817	0.035770518	Assembly of collagen fibrils and other multimeric structures	COL4A3, COL4A4	2.386494615

FDR: false discovery rate
